# Determining Information on Cardiology Disease Risk Factors of Disease in Women

**DOI:** 10.1155/2014/276121

**Published:** 2014-07-03

**Authors:** Hasret Yalçınöz Baysal, Sonay Bilgin, Işın Cantekin, Gökhan Bilgin

**Affiliations:** ^1^Department of Medical Nursing, Health Sciences Faculty, Ataturk University, Erzurum, Turkey; ^2^Department of Internal Medical Nursing, Faculty of Health Sciences, Meliksah University, Mevlana Mahallesi, Aksu Sokak, 38280 Talas, Kayseri, Turkey; ^3^Public Health Agency, Erzurum, Turkey

## Abstract

In an attempt to decrease the rate of mortality from cardiovascular diseases, first cardiovascular risk factors should be controlled. This study was carried out to reveal the level of knowledge about cardiovascular disease risk factors in women who presented to a primary healthcare center in Erzurum, Turkey, and the prevalence of such risk factors. Our study is a descriptive one and its data were collected between February and April 2013. The study included 168 women who presented to Filiz Dolunay Family Healthcare Center and who met the criteria and agreed to participate in the study. It was found that 22.6% of the women did not exercise at all above normal range, 53.6% of them had weights above normal, 23.8% smoked, and 22% had hypertension, type 2 diabetes, cholesterol, or excess fat around their waist. It was concluded that, although women were knowledgeable, they did not put this into practice in their lives.

## 1. Introduction

Cardiovascular diseases (CVD) play an increasingly important role as the major cause of mortality and morbidity and are one of the leading health issues across the world [1–3].

In our country, deaths associated with cardiovascular diseases (CVD) are at the top of the list. In an attempt to decrease the rate of mortality from cardiovascular diseases, cardiovascular risk factors should be brought under control. Economic transformation, urbanization, industrialization, and globalization inducing life style changes such as tobacco use, physical inactivity, and unhealthy diet are strictly correlated with the progression of heart diseases. Life expectancy rises rapidly in developed and developing countries and people are exposed to these risk factors for a longer time [4].

Increased rates of obesity, metabolic syndrome, and smoking in women today lead to a significant rise in morbidity and mortality associated with cardiovascular diseases. Turkish women in particular demonstrate much more negative characteristics than men in terms of some risk factors [5].

Among the cardiovascular risk factors, age, gender, and genetic/ethnical factors are in the “unchangeable factors” group. Cigarettes and other tobacco products, unhealthy eating habits, sedentary living, being overweight, dyslipidemia, hypertension, and diabetes are in the main “modifiable” cardiovascular risk factors group [1].

Individuals can lower their cardiovascular disease risk by engaging in regular physical activity, avoiding tobacco use and passive smoking, choosing a fruit and vegetable rich diet, avoiding food rich in fats, salt, and sugar, and maintaining a healthy body weight [1]. Health professionals should find out to what extent women are knowledgeable about the risk factors and risk reducing behaviors in CVD.

This study was carried out to reveal the level of knowledge about cardiovascular disease risk factors in women who presented to a primary healthcare center in Erzurum, Turkey, and the prevalence of such risk factors.

## 2. Methods

### 2.1. Study Design

Our study is a descriptive one and its data were collected between February and April 2013. The study included 168 women who presented to Filiz Dolunay Family Healthcare Center and who met the criteria and agreed to participate in the study. The questionnaires were completed by the investigator in 20 minutes by way of face-to-face interviews.

Criteria are as follows:

- nonhearing problems,
- nonpsychological discomfort,
- 18 women over the age.

### 2.2. Data Collection Tool

#### 2.2.1. The Cardiovascular Disease Risk Factors Knowledge Level (CARRF-KL) Scale

The Cardiovascular Disease Risk Factors Knowledge Level (CARRF-KL) Scale, which was prepared by Wagner et al., was tested for validity and reliability in Turkey by Arikan et al. [6, 7]. The first four items in the scale relate to the characteristics of CVD, avoidance of them, and age factor, 15 items question risk factors (items 5, 6, 9–12, 14, 18–20, 23–25, 27, and 28), and nine items (items 7, 8, 13, 15, 16, 17, 21, 22, and 26) question the resulting change in risk behavior. The items in the scale are presented as complete sentences which can be true or false and the participants are asked to answer these expressions as “yes,” “no,” or “do not know.” Each true response is given 1 point. The expressions in six of the sentences in the scale are false. These items are coded in reverse order as compared to the others. The highest total score that can be obtained from the scale is 28. Cronbach's alpha value of the scale is 0.768. Cronbach's alpha value of our study was 0.786.

The revised questionnaire was pretested in a pilot study held within the author's workplace and a number of minor adjustments were made to the original version.

### 2.3. Process

The questionnaires were completed by the investigator in 20 minutes by way of face-to-face interviews. The women who came to Filiz Dolunay Family Healthcare Center for primary healthcare and who met the criteria were provided with information about the study and those who agreed to take part in the study were included in the study. Data were entered into the Statistical Package for Social Sciences 15.0 for Windows (SPSS Inc., Chicago, IL, USA) and checked for accuracy. Questions relating to the Heart Belief Fact Questionnaire-2 were tallied by correct response and totaled. The scores were recorded on a Microsoft Office Excel spread sheet.

### 2.4. Review Board Approval

Only patients who voluntarily consented to participate were included in the study. Assurance was given that confidential personal information would not be disclosed. Permission to conduct the study was obtained from the Health Sciences Institute Ethics Committee.

## 3. Results

The ages of the women who took part in the study ranged between 30 and 75. The percentages of the true responses given by women to the questions are shown in Table 1.

The heart disease risks women have are shown in Table 2. It was found that 22.6% of the women did not exercise, 53.6% of them had weights above normal, 23.8% smoked, and 22% had hypertension, type 2 diabetes, cholesterol, or excess fat around their waist.

The women were classified with respect to heart disease and knowledge level in Table 3. There were 24 women with “0” risk factor, 32 women with “1” risk factor, 44 women with “2” risk factors, 40 women with “3” risk factors, and 28 women with “4” risk factors.

The mean scores obtained by the women from the scale were 18 ± 26 in those with “0” risk factor, 16 ± 24 in those with “1” risk factor, 12 ± 20 in those with “2” risk factors, 10 ± 18 in those with “3” risk factors, and 10 ± 16 in those with “4” risk factors, the mean total score being 10 ± 26.

## 4. Discussion

Although heart diseases have a significant place among the death rates in female population in our country, very few studies have been performed in relation to this issue. We tried in this study to determine how knowledgeable women were in heart diseases and to what extent they put their knowledge into practice.

The mean score the women obtained from the Cardiovascular Disease Risk Factors Knowledge Level was found to be 10 ± 26 in the study. Although the women had moderate levels of knowledge (the total score ranged between 0 and 28), they had one or two risk factors for heart diseases. Other studies that have been conducted on the subject support this study [8–10].

It was found in the Turkish Population and Health Survey (TNSA-2008) that 34.4% of women in the 15–49 age group were overweight and 23.9% of them were obese [11]. It was also stated that the prevalence of obesity ranged between 24.6% and 61% in women and it was twice that in men [12–14].

Approximately 60–85% of adults are not active enough to be healthy [15].

In a study made by the Ministry of Health, the percentage of those who practiced regular physical activity across the country was as low as 3.5%. In other words, 96.5% of the nation fails to practice regular physical activity [16]. It was found in another study that 20.3% of all individuals lead a sedentary life and 16% of them practiced insufficient physical activity [17]. Our study results are in parallel with these studies assessing physical activity habits and show that physical activity has not yet become a life style in our country.

According to the results of another study conducted in our country, 22.8% of the sample, 51.1% of which consisted of women, was in the obese category, 36.8% were overweight, and 34.1% had a normal body mass index and the percentage of those who did not exercise in the whole sample was 62.8% [18]. In their study, Gocgeldi et al. found that the patients who were overweight according to their body mass indexes had significantly lower scores from the general health scale [19].

Prevention in the setting of heart diseases requires a proper life style. Rosenberg et al. [20] and Kawachi et al. [21] found in their studies that female patients who smoked had a higher risk of developing heart diseases.

Mahanonda et al. investigated in their studies the effect of regular exercising on cardiovascular risk factors and found that the resting heart rate was quite lower in the group not exercising regularly than in the group exercising regularly [22]. Huxley et al. found in their study that women with diabetes had higher risk of having a heart disease than men [23]. Sharrett et al. found in their study that high LDL and low HDL were associated with CVD [24]. It has been reported that the probability of having hypertension was threefold in the obese [25].

We found in our study that each of the women had at least two risk factors for heart diseases. This result suggests that awareness is low in women and they do not put the knowledge they have into practice in their lives. The percentage of women who have a risk level of 4 (17%) is nothing to be overlooked. It can be said that these risk factors increase because people in this region eat more saturated fat and animal food and lead a sedentary life due to long and snowy winter months.

In heart diseases, an early intervention may be helpful in preventing the progression of the disease to a large extent. In a study, a program for protection from heart diseases that was administered to individuals was very beneficial in preventing the disease [26]. This shows the significance of risk assessment and trainings in heart diseases.

In conclusion, it was seen that women had the knowledge but did not practice it in their lives. Screening women for CVD risk and informing them accordingly as well as identifying those with higher CVD risk and monitoring them are important in terms of protection from CVD, early diagnosis, and treatment. Since individuals' awareness of being at risk will enhance participation in screening programs, nurses should determine risk levels of women through trainings they will organize and should share the results with them. Nurses can increase awareness by providing training to individuals.

*Limitations and Generalizability of the Study*. The fact that the study was conducted in a primary healthcare center in the province of Erzurum, Turkey, is the limitation of this study. Therefore, the results obtained in the study may be generalized only for this study group.

*Recommendations*. As multidisciplinary team members, nurses can develop programs to protect individuals from heart diseases by making use of this study. They can organize seminars enriched with written and visual materials to enlighten people. Risk factors specific to women in heart diseases (e.g., menopause, depression, and thyroid disorders) can be emphasized. Additionally, screening people for CVD risk, informing them accordingly, and monitoring them are important in terms of protection from CVD, early diagnosis, and treatment.

## Figures and Tables

**Table 1:** Responses to Heart Disease Fact Questionnaire.

Question	Correct response	
	(*N* = 168)	*n*%
A person can always realize that he/she has a heart disease.	110	65.4
Having heart disease in your family increases your risk of having a heart disease.	100	59.5
The elderly have a higher risk of heart disease.	145	86.3
Coronary heart disease can be prevented.	92	54.7
The avoidable cause of deaths and diseases in our country is smoking.	132	78.5
Smoking is a risk factor for heart disease.	146	86.9
If one quits smoking, risk of developing a heart disease diminishes.	144	85.7
Eating 2-3 fruits and 2 servings of vegetable dishes a day is beneficial.	158	94
Consuming red meat more than 3 times a week is harmful.	69	41
Salty food provokes hypertension.	159	94.6
Fatty foods do not elevate the cholesterol level in blood.	151	89.8
Fats that are solid in room temperature are good for heart health.	98	58.3
Eating a diet low in fats and carbohydrates is beneficial for the heart.	109	64.8
Overweight people have higher risk of heart disease.	134	79.7
Regular exercise lowers heart disease risk.	147	87.5
Risk is reduced only through exercise in gymnasia.	104	61.9
Slow walk and strolling around can be considered as exercise.	90	53.5
Stress, grief, and sadness increase risk of heart disease.	107	63.9
Human body increases the blood pressure in stressful situations.	143	85.1
Hypertension is a risk factor for heart disease.	154	91.6
Keeping blood pressure under control reduces the risk of developing a heart disease.	134	79.7
Antihypertensive drugs should be used lifelong.	105	62.5
High cholesterol is a risk factor for heart disease.	132	78.5
If good cholesterol (HDL) is high, there is risk of heart disease.	65	38.6
If bad cholesterol (LDL) is high, there is risk of heart disease.	93	55.3

Medication is given to everybody who has high cholesterol.	78	46.4
Diabetes is a risk factor for heart disease.	87	51.7
If sugar is kept under control in diabetic patients, risk decreases.	96	57.1

**Table 2:** Heart disease risk factor.

Variable	Number	Percent
Hypertension	37	22
Diabetes mellitus type 2	37	22
Overweight	90	53.6
Dyslipidemia	37	22
Smoking	40	23.8
Lack of exercise	38	22.6
Abdominal adiposity	37	22

**Table 3:** The mean values of the risk factors of cardiovascular disease.

Total risk factors	*N*	Mean	Minimum–maximum
0	24	19.3	18 ± 26
1	32	21.5	16 ± 24
2	44	15.6	12 ± 20
3	40	19.1	10 ± 18
4	28	17.6	10 ± 16

Total	168	20.2	10 ± 26
